# Benefit of glucosyl Hesperidin in patients with primary biliary cholangitis: A multicenter, open-label, randomized control study

**DOI:** 10.1097/MD.0000000000032127

**Published:** 2022-12-02

**Authors:** Kei Moriya, Kiyoshi Asada, Shota Suzuki, Masahide Enomoto, Yukihisa Fujinaga, Yuki Tsuji, Tadashi Namisaki, Hitoshi Yoshiji

**Affiliations:** a Department of Gastroenterology and Hepatology, Nara Medical University, Kashihara, Nara, Japan; b Institute for Clinical and Translational Science, Nara Medical University, Kashihara, Nara, Japan.

**Keywords:** gamma-glutamyl transferase, glucosyl hesperidin, nuclear factor erythroid 2-related factor 2, oxidative stress, primary biliary cholangitis

## Abstract

Globally, the number of patients with primary biliary cholangitis (PBC) is increasing. Growing evidence suggests that oxidative stress plays a significant role in the pathogenesis of chronic liver disease regardless of its etiology. Hesperidin, a natural antioxidative substance derived from citrus peel, has been shown to have an anti-inflammatory effect in a rat arthritis model and may be a potential substance to attenuate intrahepatic inflammation in patients with PBC. In this study, the potential of glucosyl hesperidin as a therapeutic agent for PBC will be investigated through antioxidative stress mechanisms.

**Methods:**

Patients with PBC who are 20 years or older will be eligible to participate. Patients will be assigned to 1 of 2 groups and given either 500 or 1000 mg of glucosyl hesperidin per day. The primary endpoint is the ratio of changes in serum gamma-glutamyl transferase levels before and after 24 weeks of glucosyl hesperidin administration. The secondary endpoints are serum hepatobiliary enzyme levels (alkaline phosphatase, transaminase, and total bilirubin levels) and the protein expression levels of nuclear factor erythroid 2-related factor 2 and its target molecule 8, 16, and 24 weeks after administration compared to before administration.

**Discussion:**

The prospective clinical interventional study was designed to assess the supportive effect of glucosyl hesperidin on hepatic function in patients with PBC receiving basic ursodeoxycholic acid treatment.

## 1. Introduction

In Japan, the number of patients with chronic liver disease has risen to 9 million for chronic hepatitis and 0.4 to 0.5 million for liver cirrhosis.^[1]^ The etiology of liver cirrhosis is changing, owing primarily to the establishment of chronic hepatitis C treatment, and the proportion of non-viral liver diseases is actually increasing. In fact, the number of patients with autoimmune hepatitis and/or primary biliary cholangitis (PBC) has more than tripled in the last decade.^[2]^ Even though treatments for viral hepatitis have made significant progress, the pathophysiology of autoimmune hepatitis and PBC remains unknown, and no curative treatment has been established.

Growing evidence suggests that oxidative stress plays a significant role in the pathogenesis of chronic liver disease regardless of the cause of liver damage.^[3–7]^ Furthermore, in patients with PBC, a mechanism that impairs antioxidant activity via the nuclear factor erythroid 2-related factor 2 (Nrf2)/Kelch-like ECH-associated protein 1 pathway was reported.^[8]^ However, it is unknown whether activating the Nrf2/Keap1 pathway improves PBC pathology.

Ursodeoxycholic acid (UDCA) is widely used as a basic therapeutic agent for PBC and has been reported to promote hepatic Nrf2 activation and reduce oxidative stress.^[9]^ However, because intact enterohepatic circulation is required for its medicinal effect, its effect is significantly attenuated in the pathological condition in which cholestasis has progressed when compared to the early stage of the disease.^[10]^ Previous research has shown that oxidative stress is an independent predictor of stage progression in patients with PBC^[11]^ and that antioxidant levels of vitamins A, C, and E are significantly lower.^[12]^ Taking these facts into account, Nrf2 activation was linked to an improvement in the pathological condition of PBC.

Glucosyl hesperidin is a type of water-soluble polyphenol with high component stability among antioxidants, and its effect is expected to be sufficient. It has been certified by Japanese Agricultural Standards as a safe anti-whitening agent for canned oranges or orange juice. Glucosyl hesperidin is a water-soluble derivative of hesperidin, and hesperidin was found in sera hydrolyzed with beta-glucuronidase after the oral administration of glucosyl hesperidin.^[13]^ Serum hesperidin levels increase in a dose-dependent manner without causing any adverse events (AEs), and human blood levels have been reported to be approximately 12 µM in the case of a 3000 mg oral intake.^[14]^ In this study, hesperidin was effective when administered with standard anti-rheumatoid therapy in ameliorating rheumatoid arthritis in mice and humans without any adverse effects. In a rat study, hesperidin oral administration resulted in concentration-dependent activation of Nrf2 in the liver and improvement of the inflammatory index.^[15]^ Additionally, organ protective effects of hesperidin on liver and spleen were also rereported.^[16–18]^

By the way, there have been many reports which show that improvement over time in biochemical test values such as serum gamma-glutamyl transferase (GGT) and alkaline phosphatase after medical therapy intervention is very useful as a better indicator of long-term life prognosis in patients with PBC.^[19–22]^

Based on these evidences, the potential of glucosyl hesperidin as a therapeutic agent for PBC will be investigated in this study through antioxidative stress mechanisms by evaluating the improvements of hepatic biochemical indicis including GGT and alkaline phosphatase.

## 2. Methods

### 2.1. Design

**Figure 1.**
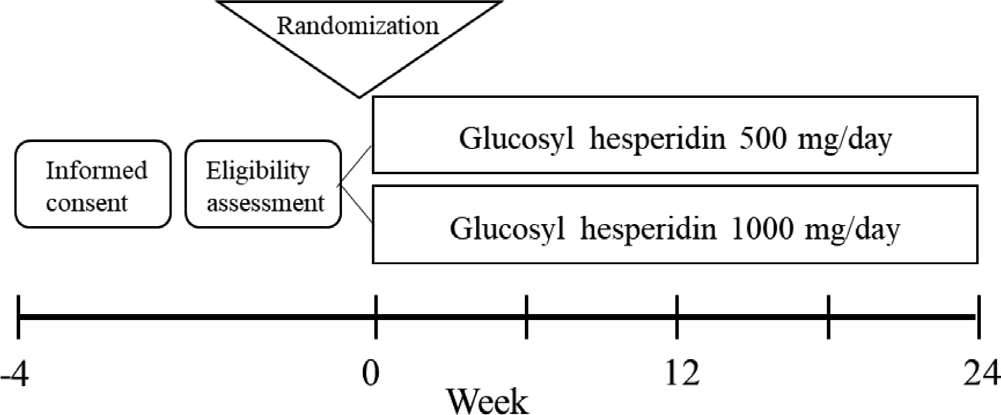
Study design. A total of 110 patients with PBC (55 in each group) will participate in this study. Following eligibility confirmation, patients will be randomly assigned to either the hesperidin 500 mg or 1000 mg treatment groups by an envelope method.

This is a multicenter, open-label, randomized, parallel-group study comparing the influence of glucosyl hesperidin on liver function with UDCA treatment in patients with PBC (Fig. 1). Patients aged 20 years or older are eligible to participate. The inclusion and exclusion criteria are listed in Table 1. Patients will be randomly assigned to either the 500 mg/day or 1000 mg/day treatment groups. In these treatment groups, either 500 mg or 1000 mg of glucosyl hesperidin will be given orally once per day for 24 weeks in addition to ongoing UDCA treatment.

**Table 1:** Inclusion and exclusion criteria.

Inclusion criteria
1.Primary biliary cholangitis.
2.Be at least 20 years old at the time of consent acquisition.
3.Patients with a performance status ECOG of 0 or 1.
4.Patients with no dose changes for UDCA and fibrate.
5.Patients who were adequately informed about the study’s objectives and voluntarily provided written consent by themselves or their surrogate.
Exclusion criteria
1.Patients with hesperidin hypersensitivity.
2.Patients who routinely consume hesperidin-containing health foods prior to participating in this study.
3.Patients scheduled for or undergoing treatment for malignant tumors.
4.Patients with a NYHA functional classification of 2 or higher.
5.Patients who experience dyspnea during daily exertion.
6.Patients with a total bilirubin level of 3.0 mg/dL or higher, or a Child-Pugh classification of B or C.
7.Patients with an eGFR of less than 30 mL/minute/1.73 m^2^.
8.Patients who are or may become pregnant.
9.Patients who participated in other clinical trials within 3 months before starting hesperidin administration.
10.Patients deemed unsuitable by doctors to participate as research subjects.

ECOG = Eastern cooperative oncology group, eGFR = estimated glomerular filtration rate, NYHA = New York heart association, UDCA = ursodeoxycholic acid.

This study will be carried out in accordance with the Helsinki declaration (revised in October 2013) and the Clinical Trials Act of the Japanese Ministry of Health, Labour, and Welfare. The investigator will provide each patient with a thorough explanation of the study and will obtain written informed consent. The study protocol was approved by a certified ethics committee. The research will last from March 30, 2022, to March 31, 2024. The study’s organization is shown in jRCTs051210210.

### 2.2. Randomization

The study participants will be registered and allocated using stratified permuted block randomization. Participants will be randomly assigned to either the 500 mg/day or 1000 mg/day groups based on the following factors: registered facility and patient sex. Table 2 provides an overview of all visit and test schedules.

**Table 2:** An overview of observation, clinical examination, and evaluation.

	Screening within 1 month	Obtaining informed consent within 1 month	Observation period					
			Treatment start date	8 weeks	16 weeks	24 weeks	36 weeks	At the time of discontinuation
Permissible range (weeks)				±2	±2	±2	±4	
Visits		1^st^	2^nd^	3rd	4th	5th	6th	
Allocation		✓						
Patient’s characteristics	✓	✓						
Treatment								
Adherence check			✓	✓	✓	✓		
Subjective symptoms	✓	✓	✓	✓	✓	✓	✓	✓
Adverse events			✓	✓	✓	✓	✓	✓
Blood examination			✓^*1^	✓	✓	✓	✓	✓^*2^

✓ means the day for each event in left column. *1: This examination can be omitted if a blood test is performed during the screening period or consent is obtained. *2: This examination can be omitted if patient consent cannot be obtained.

AEs = adverse events.

### 2.3. Endpoints

The primary endpoint is the rate of change in serum GGT levels from 0 to 24 weeks after glucosyl hesperidin administration. AEs will be collected for safety analysis. Any unfavorable or unexpected sign, symptom, or disease, including abnormal laboratory results, is considered an AE. Patients who have had their safety evaluation data collected after the start of study treatment will be included in the safety analysis set. The full analysis set and per-protocol set will be used to assess efficacy. The full analysis set is defined as the patient population that was excluded from the safety analysis set for any of the following reasons: ineligible cases after enrollment; and cases with no efficacy data at any time after the start of study treatment.

### 2.4. Sample size

Since PBC, the study’s target disease, is a rare disease, the total number of participants in the study’s participating facility is expected to be around 110, and it is estimated to be 100 after the dropout. The difference between the treatment groups (standard and high dose administration groups) from the start (0 weeks) to the end of administration (24 weeks) is 5%, and the standard deviation is 5%. With a 7.5% probability, a power of 90% can be obtained at a significance level (both sides) of 5%.

### 2.5. Statistical analysis

#### 2.5.1. Collection of background (baseline) data.

The background (baseline) data of the research subjects will be tabulated. For nominal and ordinal variables, the frequency and proportion of categories will be calculated. For continuous variables, summary statistics such as mean, standard deviation, minimum, median, and maximum will be calculated. Values of *P* < .05 will be considered as statistical significance. JMP version 14.3 (SAS Institute Inc, Cary, NC) software and EZR software, which is a GUI for the R statistical computing and graphics environment,^[23]^ will be used for statistical analyses.

#### 2.5.2. Primary endpoint.

The average “rate of change of biliary enzyme levels (serum GGT levels) at 24 weeks after glucosyl hesperidin administration from before administration (0 weeks)” will be compared between groups using an unpaired Student’s *t* test. In addition, the point estimate of the difference between the groups and the 95% confidence interval will be calculated.

The rate of change will be calculated as follows: serum GGT level (24 weeks)-serum GGT level (0 weeks)/serum GGT level (0 weeks).

#### 2.5.3. Secondary endpoint.

For each item, the mean values for each group will be compared between groups using an unpaired Student’s *t* test. In addition, the point estimate of the difference between the groups and the 95% confidence interval will be calculated. The rate of change will be calculated as follows: each evaluation item (at each evaluation point other than 0 weeks) – each evaluation item (0 weeks)/each evaluation item (0 weeks). The following items will be included: Rate of change in biliary enzyme levels (serum GGT level) after glucosyl hesperidin administration (8 and 16 weeks) from before administration (0 weeks); rate of change in hepatobiliary enzyme levels (serum alkaline phosphatase, transaminase, and total bilirubin levels) after glucosyl hesperidin administration (8, 16, and 24 weeks) from before administration (0 weeks); and rate of change in Nrf2 protein expression levels and its target gene (HMOX-1) after glucosyl hesperidin administration (8, 16, and 24 weeks) from before administration (0 weeks).

#### 2.5.4. Exploratory endpoint.

For each item, the mean values for each group will be compared between groups using an unpaired Student’s *t* test. In addition, the point estimate of the difference between the groups and the 95% confidence interval will be calculated. The rate of change will be calculated as follows: each evaluation item (at each evaluation point other than 0 weeks)-each evaluation item (0 weeks)/each evaluation item (0 weeks).

The following items will be included: The rate of change in the protein expression level of inflammatory cytokines (tumor necrosis factor-alpha, interleukin 6, and IL-1β), as well as peroxisome proliferator-activated receptor-gamma (peroxisome proliferator-activated receptor-γ), superoxide dismutase, and glutathione peroxidase, which have an inhibitory effect on the activation of the inflammation/immune-related transcription factor nuclear factor-kappa B before and after glucosyl hesperidin administration (0–8, 0–16, and 0–24 weeks).

#### 2.5.5. Other analysis items.

The ratio for each research subject and the average will be calculated to determine adherence. In particular, the previous dose intake rate corresponds to: 70% or more, 30% or more and less than 70%, or less than 30% for each visit during the period of glucosyl hesperidin administration.

#### 2.5.6. Safety analysis.

All AEs will be evaluated according to the Common Terminology Criteria for Adverse Events version 5.0 proposed by National Cancer Institute. The incidence of each AE will be calculated in the glucosyl hesperidin-administered subjects during the study’s observation period. The period for collecting data related to all evaluation items will be completed for all analyses, and the analysis will be performed once the data is fixed.

#### 2.5.7. Data monitoring.

Serum levels of GGT, alkaline phosphatase, transaminase, and total bilirubin will be measured in the clinical laboratories in each facility. Those of tumor necrosis factor-alpha, interleukin 6, IL-1β, peroxisome proliferator-activated receptor-γ, superoxide dismutase, glutathione peroxidase, Nrf2, and HMOX-1 will be measured by the authors using the commercially available enzyme-linked immunosorbent assay kit. All data will be stored in a database in a locked cabinet at the laboratory of the Department of Gastroenterology and Hepatology at Nara Medical University. The assessors will enter the data into the database system. The trial enrollment and duration will be limited to ensure data robustness.

#### 2.5.8. The quality and safety monitoring.

The trial’s safety will be monitored once a month by an independent physician with relevant expertise. All participants and visits will be monitored. On the other hand, the auditors will inspect the process several times throughout the period.

#### 2.5.9. Confidential.

All personal information about potential and enrolled participants, as well as proxies, will be kept in a secure location to which no third party will have access.

## 3. Discussion

This study was designed to assess whether glucosyl hesperidin has a positive effect on hepatic function in patients with PBC. Patients with PBC, who are mostly middle-aged and older women, have a relatively high prevalence of comorbidities and are generally prone to high oxidative stress conditions. In addition, previous studies have shown that antioxidant activity via the Nrf2/Keap1 pathway is more severely impaired with the exacerbation of liver pathology in patients with PBC.^[8]^ Furthermore, it has recently been reported that S-adenosyl-L-methionine, which is associated with redox regulation, protects against mitochondria-derived oxidative stress by upregulating the expression of Nrf2 and its target gene, HMOX-1, in human cholangiocytes.^[24]^ Thus, the Nrf2/Keap1 pathway is generally impaired in chronic liver diseases, including PBC, and attempts to inhibit the pathological progression of chronic liver diseases by activating this pathway are easily understood. UDCA, the first-line drug for PBC, has the ability to activate the Nrf2/Keap1 pathway. However, because enterohepatic circulation is required for its efficacy, UDCA’s pharmacological effect naturally diminishes as intrahepatic biliary stasis progresses. Therefore, new drug candidates for the treatment of PBC must include ingredients that are safe to use in combination with UDCA in terms of duration and dosage. In this regard, glucosyl hesperidin has been chosen as the target component of this medical intervention study, and a new treatment regimen combining UDCA, the standard treatment for PBC, with glucosyl hesperidin has been proposed, with a randomized clinical trial to confirm its efficacy. Glucosyl hesperidin is a common natural component derived from citrus plants that acts as a safe and effective Nrf2/Keap1 pathway activator.^[25]^ The concept of using an existing chemical substance with established safety for oral intake to improve the condition of a designated intractable disease whose pathology is not fully understood is novel and will contribute to the development of a new treatment option. If this study shows that glucosyl hesperidin has a dose-dependent effect on lowering serum biliary enzyme levels, it would be very significant because it would mean that intrahepatic microcholangitis in patients with PBC is being treated, and thus may contribute to the suppression of liver fibrosis progression in a physically safe manner. Furthermore, if the medical efficacy of glucosyl hesperidin in patients with PBC is confirmed, the impact of these study’s findings will be enormous, as it may lead to improvements in the pathogenesis of numerous other inflammatory diseases in which disruption of the antioxidant stress mechanism is a major etiological factor. In this study, case enrollment began in the summer of 2022, with results expected in 2024.

## Acknowledgments

We would like to thank the staff of Nara Medical University’s Gastroenterology and Hepatology, Nara Prefecture General Medical Center, and Nara Medical University Hospital’s Institute for Clinical and Translational Science, particularly Yayoi Nakamura, for their assistance in refining the protocol.

## Author contributions

KM contributed to the conceptualization. The study protocol was designed by KM; KA, and SS. The main manuscript text was written by KM and KA. The manuscript was reviewed by ME, YF, YT, TN, and HY. All authors read and approved the final manuscript.

## References

[1] Current report on primary liver cancer in Japan 2015 (In Japanese). Available at: https://www.jsh.or.jp/lib/files/medical/guidelines/jsh_guidlines/liver_cancer_2015.pdf. Published by Japan Society of Hepatology. [access date September 30, 2022].

[2] Tanaka A Mori M Matsumoto K. Increase trend in the prevalence and male-to-female ratio of primary biliary cholangitis, autoimmune hepatitis, and primary sclerosing cholangitis in Japan. Hepatol Res. 2019;49:881–9. 30932290 10.1111/hepr.13342

[3] White AM Orosz A Powell PA. Alcohol and aging-an area of increasing concern. Alcohol. 2022:S0741–8329(22)00066-0. 10.1016/j.alcohol.2022.07.005 35940508

[4] Suhail M Sohrab SS Kamal MA. Role of hepatitis C virus in hepatocellular carcinoma and neurological disorders: an overview. Front Oncol. 2022;29:913231. 10.3389/fonc.2022.913231 PMC9372299 35965577

[5] Usman M Bakhtawar N. Vitamin E as an adjuvant treatment for non-alcoholic fatty liver disease in adults: a systematic review of randomized controlled trials. Cureus. 2020;12:e9018. 32775098 10.7759/cureus.9018 PMC7405968

[6] Sadasivam N Kim YJ Radhakrishnan K. Oxidative stress, genomic integrity, and liver diseases. Molecules. 2022;27:3159. 35630636 10.3390/molecules27103159 PMC9147071

[7] Simicic D Cudalbu C Pierzchala K. Overview of oxidative stress findings in hepatic encephalopathy: from cellular and ammonium-based animal models to human data. Anal Biochem. 2022;654:1147954795. 10.1016/j.ab.2022.114795 35753389

[8] Wasik U Milkiewicz M Podhorodecka AK. Protection against oxidative stress mediated by the Nrf2/Keap1 axis is impaired in Primary Biliary Cholangitis. Sci Rep. 2017;7:44769. 28333129 10.1038/srep44769 PMC5363061

[9] Kawata K Kobayashi Y Souda K. Enhanced hepatic Nrf2 activation after ursodeoxycholic acid treatment in patients with primary biliary cirrhosis. Antioxid Redox Signal. 2010;13:259–68. 20055754 10.1089/ars.2009.2903

[10] Grattagliano I Palmieri VO Portincasa P. Long-term ursodeoxycholate improves circulating redox changes in primary biliary cirrhotic patients. Clin Biochem. 2011;44:1400–4. 21963381 10.1016/j.clinbiochem.2011.09.008

[11] Sorrentino P Terracciano L D’Angelo S. Oxidative stress and steatosis are cofactors of liver injury in primary biliary cirrhosis. J Gastroenterol. 2010;45:1053–62. 20393861 10.1007/s00535-010-0249-x

[12] Cash WJ McCance DR Young IS. Primary biliary cirrhosis is associated with oxidative stress and endothelial dysfunction but not increased cardiovascular risk. Hepatol Res. 2010;40:1098–106. 20977566 10.1111/j.1872-034X.2010.00717.x

[13] Yamada M Tanabe F Arai N. Bioavailability of glucosyl hesperidin in rats. Biosci Biotechnol Biochem. 2006;70:1386–94. 16794318 10.1271/bbb.50657

[14] Kometani T Fukuda T Kakuma T. Effects of alpha-glucosylhesperidin, a bioactive food material, on collagen-induced arthritis in mice and rheumatoid arthritis in humans. Immun Immunotoxicol. 2008;30:117–34. 10.1080/08923970701812688 18306109

[15] Ahmed YM Messiha BA Abo-Saif AA. Protective effects of simvastatin and hesperidin against complete freund’s adjuvant-induced rheumatoid arthritis in rats. Pharmacol. 2015;96:217–25. 10.1159/000439538 26345515

[16] Hanchang W Wongmanee N Yoopum S. Protective role of hesperidin against diabetes induced spleen damage: mechanism associated with oxidative stress and inflammation. J Food Biochem. 2022:e14444. 36165434 10.1111/jfbc.14444

[17] Shimamura Y Sei S Nomura S. Protective effects of dried mature citrus unshiu peel (Chenpi) and hesperidin on aspirin-induced oxidative damage. J Clin Biochem Nutr. 2021;68:149–55. 33879966 10.3164/jcbn.20-83 PMC8045996

[18] Eldin DN Fahim HI Ahmed HY. Preventive effects of mandarin fruit peel hydroethanolic extract, hesperidin, and quercetin on acetaminophen-induced hepatonephrotoxicity in Wistar rats. Oxid Med Cell Longev. 2022;2022:7065845. 36092164 10.1155/2022/7065845 PMC9463012

[19] Carbone M Sharp SJ Flack S. The UK-PBC risk scores: derivation and validation of a scoring system for long-term prediction of end-stage liver disease in primary biliary cholangitis. Hepatol. 2016;63:930–50. 10.1002/hep.28017 PMC6984963 26223498

[20] Lammers WJ Hirschfield GM Corpechot C. Development and validation of a scoring system to predict outcomes of patients with primary biliary cirrhosis receiving ursodeoxycholic acid therapy. Gastroenterol. 2015;149:1804–12.e4. 10.1053/j.gastro.2015.07.061 26261009

[21] Azemoto N Kumagi T Abe M. Biochemical response to ursodeoxycholic acid predicts long-term outcome in Japanese patients with primary biliary cirrhosis. Hepatol Res. 2011;41:310–7. 21426448 10.1111/j.1872-034X.2011.00782.x

[22] Namisaki T Moriya K Noguchi R. Liver fibrosis progression predicts survival in patients with primary biliary cirrhosis. Hepatol Res. 2017;47:E178–86. 27189879 10.1111/hepr.12746

[23] Kanda Y. Investigation of the freely available easy-to-use software “EZR” for medical statistics. Bone Marrow Transplant. 2013;48:452–8. 23208313 10.1038/bmt.2012.244 PMC3590441

[24] Kilanczyk E Banales JM Wunsch E. S-adenosyl-L-methionine (SAMe) halts the autoimmune response in patients with primary biliary cholangitis (PBC) via antioxidant and S-glutathionylation processes in cholangiocytes. Biochim Biophys Acta Mol Basis Dis. 2020;1866:165895. 32681864 10.1016/j.bbadis.2020.165895

[25] Mahmoud AM Mohammed HM Khadrawy SM. Hesperidin protects against chemically induced hepatocarcinogenesis via modulation of Nrf2/ARE/HO-1, PPARγ and TGF-β1/Smad3 signaling, and amelioration of oxidative stress and inflammation. Chem Biol Interact. 2017;277:146–58. 28935427 10.1016/j.cbi.2017.09.015

